# Cultural Adaptation and Structural Validation of the Short-Form Örebro Musculoskeletal Screening Questionnaire in Peruvian Nurses with Musculoskeletal Symptoms

**DOI:** 10.3390/healthcare14142044

**Published:** 2026-07-08

**Authors:** Isaac Bardales-Caman, Pamela Asto-Campos, Mardel Morales-García, Daniel W. Richard-Pérez, Wilter C. Morales-García, Liset Z. Sairitupa-Sanchez

**Affiliations:** 1 Unidad de Salud Pública, Escuela de Posgrado, Universidad Peruana Unión, Lima 15102, Peru; 2 Facultad de Teología, Universidad Peruana Unión, Lima 15102, Peru; wiltermorales@upeu.edu.pe; 3 Escuela de Psicología, Universidad Señor de Sipán, Chiclayo 14001, Peru

**Keywords:** Peruvian nurses, validation, musculoskeletal screening, confirmatory factor analysis

## Abstract

**Introduction:** Work-related musculoskeletal disorders are common among nursing staff and may affect functionality, psychological well-being, and work performance. Brief, culturally adapted, and psychometrically sound instruments are needed to characterize musculoskeletal risk in occupational settings. This study aimed to translate, culturally adapt, and examine the structural validity and internal reliability of the abbreviated version of the Örebro Musculoskeletal Screening Questionnaire in Peruvian nurses with musculoskeletal symptoms. **Methods:** An instrumental, cross-sectional study was conducted with a sample of 170 Peruvian nurses. The Spanish-adapted version of the ÖMSQ-12S was administered. Descriptive statistics, corrected item-total correlations, confirmatory factor analysis using the robust maximum likelihood estimator, internal reliability through Cronbach’s alpha and composite reliability, convergent validity through average variance extracted, and discriminant validity using the Fornell–Larcker criterion were analyzed. **Results:** The original three-dimensional 12-item model showed inadequate fit: χ^2^(51) = 276.301, CFI = 0.713, TLI = 0.628, RMSEA = 0.161, and SRMR = 0.190. Due to low and divergent factor loadings, a refined 11-item, four-factor solution was evaluated: functionality/satisfaction, fear-avoidance, psychological aspects, and characteristics of the musculoskeletal problem. This model showed better fit: χ^2^(37) = 58.190, CFI = 0.971, TLI = 0.957, RMSEA = 0.058, and SRMR = 0.039. Reliability was adequate across all factors (α = 0.773–0.893; CR = 0.779–0.895). Convergent validity was acceptable in most dimensions, although discriminant validity was limited between psychological aspects and characteristics of the musculoskeletal problem. **Conclusions:** The ÖMSQ-11S showed preliminary evidence of structural validity and internal reliability in Peruvian nurses with musculoskeletal symptoms. However, its use as a predictive tool or universal occupational screening instrument requires further studies with longitudinal designs, more heterogeneous samples, and external clinical or occupational criteria.

## 1. Introduction

Work-related musculoskeletal disorders (WMSDs) are among the leading emerging threats to occupational health in the twenty-first century because of their high prevalence, chronic nature, and debilitating consequences at both the individual and institutional levels [1,2]. These conditions, which affect muscles, bones, tendons, ligaments, joints, and nerves, are caused by prolonged exposure to physical risk factors (awkward postures, manual handling of loads), ergonomic factors (repetitive movements, excessive exertion), and psychosocial factors (stress, overload, rotating shifts) in the workplace [3]. The World Health Organization (WHO) and the International Labour Organization (ILO) have recognized WMSDs as a “new epidemic” in occupational health, urging health systems to adopt urgent prevention and intervention measures [2]. In this context, nursing staff emerge as one of the most affected occupational groups. Multiple studies have documented alarming prevalences of WMSDs among nurses, with rates ranging from 60% to 98%, with low back pain, neck pain, and upper-limb symptoms being the most frequent manifestations [4,5]. Recent studies in Europe report that nearly 88% of nurses experience at least one WMSD over the course of their careers, while in China the prevalence reaches 79%, with higher rates among women and professionals with more than 15 years of service [6,7]. These figures reflect not only the global magnitude of the problem but also differential patterns according to age, gender, service area, and work history, underscoring the need for contextually adapted approaches. The causes are multifactorial, ranging from physically demanding tasks such as patient mobilization to long working hours and constant exposure to psychosocial stress [8,9]. These conditions are exacerbated by staff shortages, shift rotation, the aging of the workforce, and insufficient institutional ergonomic policies [10,11].

The consequences of WMSDs among nurses extend beyond the physical domain. In addition to health deterioration, these conditions are associated with poorer quality of life, symptoms of anxiety and depression, absenteeism, and an increasing intention to leave the profession, thereby worsening the already critical shortage of human resources in health care [12,13]. The economic impact is also considerable: a single musculoskeletal injury is estimated to generate costs exceeding USD 50,000, including medical care, compensation, and productivity losses [5]. From an institutional perspective, WMSDs among nursing staff represent a direct threat to the continuity and quality of patient care. Nurses with chronic pain show higher rates of errors, reduced timely care, and poorer teamwork, affecting the safety of the clinical environment [14,15]. In low- and middle-income countries, where the care burden is greater and resources are limited, these consequences are further aggravated, as documented in contexts such as Nepal, Ecuador, and Brazil [16,17].

Individual factors also play a significant role in the incidence of WMSDs. Variables such as age, body mass index (BMI), gender, work experience, and emotional state modulate workers’ vulnerability to developing these conditions [18,19]. In particular, young workers and those without ergonomic training present early symptoms, whereas older workers face a reduced capacity for recovery [20,21]. In addition, meta-analytic studies have confirmed that organizational factors such as low institutional support, high task demands, and job dissatisfaction are strongly associated with the onset of WMSDs [22,23].

Given this scenario, the implementation of comprehensive prevention programs that combine ergonomic strategies, active breaks, occupational health education, and improvements in working conditions is essential to protect nurses’ health and ensure the sustainability of the health care system [24,25]. Early identification of workers at risk of developing chronic disability due to musculoskeletal conditions is essential, not only because of its implications for nurses’ quality of life but also because of the economic impact these conditions impose on health systems, insurers, and institutional productivity [26,27]. Although only a small percentage of acute musculoskeletal injuries progress to chronicity, this subgroup accounts for the greatest financial, personal, and social burden [28,29].

Given this problem, brief musculoskeletal assessment questionnaires may serve as useful complementary tools for characterizing physical, functional, and psychosocial factors associated with musculoskeletal pain in clinical and occupational settings [30]. However, their use as predictive screening instruments requires specific empirical evidence, including criterion validity, temporal stability, and the ability to anticipate relevant outcomes such as pain persistence, functional disability, work absenteeism, or return to work. Among the available tools, the Örebro Musculoskeletal Pain Screening Questionnaire has progressively evolved to assess factors associated with the persistence of musculoskeletal problems, incorporating physical, emotional, cognitive, and functional components related to the pain experience. Consequently, before recommending its broad use in new cultural or occupational contexts, it is necessary to carefully examine its measurement properties in the specific population of interest. The first version, known as the ÖMPSQ (Örebro Musculoskeletal Pain Screening Questionnaire), was developed by Linton and Boersma in Sweden in 2003. This original version consists of 25 items, 21 of which are scored, and assesses pain, physical function, emotional factors, and beliefs about pain [31]. To expand its application to other cultural contexts, international adaptations of the ÖMPSQ were developed. In 2012, the French version was validated while retaining the original 25 items, confirming a unidimensional structure and adequate predictive capacity to distinguish among patients at low, moderate, and high risk of chronification [32]. Subsequently, in 2014, the Spanish version (OMPQ-ES), also composed of 25 items, was presented, identifying six dimensions: Pain, Coping, Emotional Distress, Return-to-Work Expectations, Fear-Avoidance, and Functionality [33]. In 2016, other relevant validations were conducted. In Hong Kong, the Chinese version (COMPSQ-HK) was developed, retaining 25 items (21 scored items and 4 demographic items) [34]. That same year, the scale was adapted in Turkey as the ÖMPQ-TR, confirming three main dimensions: psychological factors and avoidance beliefs, physical disability, and work absenteeism and job satisfaction [35]. In 2017, the Persian version (OMPQ-Persian) was published, consisting of 21 self-administered items that assess physical and psychosocial risk factors for chronic disability. This version was considered unidimensional. One year later, in 2018, the German version (OMPSQ-G), with 21 items, was presented and validated in patients with chronic neck pain. This adaptation confirmed its unidimensional structure [36]. To improve efficiency of use, the short-form version, the ÖMPSQ-SF (Örebro Musculoskeletal Pain Screening Questionnaire—Short Form), was developed in 2011. It consists of 10 items distributed across five dimensions: pain, perceived function, emotional distress, fear-avoidance beliefs, and return-to-work expectations. This version showed a high correlation with the original scale and adequate predictive capacity in different clinical contexts [37]. In 2022, the Japanese version (ÖMPSQ-SF-JP) was validated in patients with chronic low back pain [38]. More recently, in 2023, a longitudinal study was conducted in Finland using the ÖMPSQ-SF, further supporting its utility as a population-level screening tool [39]. In 2013, another brief version, the ÖMSQ-12 (Örebro Musculoskeletal Screening Questionnaire—12 items), was developed to comprehensively assess the risk of persistence of musculoskeletal problems in patients with work-related injuries. This version was reduced to 12 items distributed across three dimensions. Its design sought to optimize clinical practicality and reduce response burden without compromising the psychometric quality of the instrument [40].

Despite the diversity of available versions and adaptations, Peru still lacks a culturally adapted version of the ÖMSQ-12 with initial psychometric evidence for use among nursing staff. This gap is relevant, considering that nurses constitute an occupational population highly exposed to musculoskeletal symptoms arising from the physical, ergonomic, and psychosocial demands inherent to the health care environment. However, because the ÖMSQ-12 was originally conceived as a tool for screening and stratifying the risk of persistent musculoskeletal problems, its adaptation to new contexts requires careful examination of its measurement properties before recommending its broad use in occupational surveillance programs.

In this regard, the selection of the ÖMSQ-12 is justified by its biopsychosocial approach, brevity, and potential usefulness for describing dimensions related to physical functioning, fear avoidance, psychological aspects, and characteristics of musculoskeletal problems among symptomatic workers. Nevertheless, cultural adaptation and evaluation of the internal structure of an abbreviated version do not, by themselves, constitute sufficient evidence of predictive utility, diagnostic accuracy, or effectiveness as a universal occupational screening tool. This is especially relevant when external criteria are not included, such as work absenteeism, pain persistence, functional disability, medical leave, use of health services, or return to work. Therefore, the present study should be understood as an initial psychometric evaluation focused on the translation, cultural adaptation, internal structure, and reliability of the instrument among Peruvian nurses with musculoskeletal symptoms.

## 2. Methods

### 2.1. Design and Participants

This was an instrumental, cross-sectional study [41] aimed at the translation, cultural adaptation, and initial psychometric evaluation of the abbreviated 12-item version of the Örebro Musculoskeletal Screening Questionnaire (ÖMSQ-12). Consistent with the nature of the study, the internal structure, reliability, and evidence of convergent and discriminant validity of the instrument were examined in a symptomatic occupational sample of Peruvian nurses.

A non-probability convenience sampling method was used. The minimum required sample size was estimated using the A-priori Sample Size Calculator for Structural Equation Models developed by Daniel Soper (Fullerton, CA, USA) [42], considering the number of observed and latent variables, a small expected effect size (λ = 0.10), a significance level of α = 0.05, and statistical power of 1 − β = 0.80. Under these parameters, the estimated minimum sample size was 100 participants. However, a total of 170 nurses were recruited, with ages ranging from 19 to 66 years (M = 32.06; SD = 10.91). Nurses were included if they met the following criteria: (a) performed clinical care duties in Peruvian health care institutions; (b) presented musculoskeletal symptoms at the time of evaluation or during the study reference period; (c) had been referred or identified by a treating physician or physical therapist for evaluation of musculoskeletal complaints; and (d) voluntarily agreed to participate by providing informed consent. Participants with incomplete responses, inconsistent response patterns, or lack of informed consent were excluded. The decision to work with a symptomatic sample was based on the purpose of conducting an initial psychometric evaluation of the instrument among nurses with musculoskeletal manifestations relevant to the construct being assessed. However, this strategy implies that the results should be interpreted as initial evidence in a symptomatic occupational population and not as a definitive validation for universal screening programs among asymptomatic nurses. Therefore, generalization of the findings to broader preventive contexts requires further studies with more heterogeneous occupational samples, including personnel without musculoskeletal symptoms. Most participants were women (85.3%) and single (67.6%). Regarding educational background, 55.9% held a professional nursing degree, 22.9% had specialization studies, and 21.2% had postgraduate studies. With respect to employment status, 26.5% worked under outsourced employment arrangements, followed by CAS contracts (23.5%) and permanent appointments (21.2%). In terms of geographic origin, the Highlands region predominated (42.4%), followed by the Coast (37.1%) and the Jungle region (20.6%) (Table 1).

### 2.2. Instruments

Örebro Musculoskeletal Screening Questionnaire. The Shortened Örebro Musculoskeletal Screening Questionnaire (ÖMSQ-12), originally developed by Gabel, Burkett, and Melloh and published as “The shortened Örebro Musculoskeletal Screening Questionnaire: Evaluation in a work-injured population” [40], was used to facilitate the brief assessment of physical, psychological, and functional factors related to the persistence of musculoskeletal problems in working populations. The abbreviated version consists of 12 items selected through two reduction approaches: one based on content retention and another based on exploratory factor analysis. The content-retention version was recommended because of its stronger clinimetric properties and its ability to preserve relevant dimensions of the original instrument. The ÖMSQ-12 assesses three dimensions: (1) physical functionality, fear-avoidance, and satisfaction; (2) psychological aspects; and (3) characteristics of the musculoskeletal problem. Items are answered on an 11-point Likert-type scale, and some items require reverse scoring according to the original scoring instructions. In the original study, the abbreviated version showed adequate internal consistency by dimension and test–retest reliability. However, in the present study, only psychometric properties related to the internal structure, internal reliability, and convergent/discriminant validity of the version adapted into Peruvian Spanish were evaluated.

The Spanish translation of the ÖMSQ-12 followed a rigorous cultural adaptation process based on methodological recommendations [43], in order to ensure linguistic and conceptual equivalence with the original instrument. This process included the following stages:

- Two native Spanish-speaking bilingual translators independently translated the ÖMSQ-12 into Spanish; both versions were compared to produce a consensus version.
- This version was subsequently translated back into English by two native English speakers from the United States who were proficient in Spanish and had no prior familiarity with the questionnaire, in order to assess semantic fidelity.
- An expert committee composed of two nurses, two psychologists, and one physician reviewed the Spanish-translated version together with the back-translations to develop a preliminary version.
- Finally, this version was evaluated in a focus group composed of five nurses, who confirmed its clarity and readability. Because no comprehension difficulties were identified, no additional adjustments were made, resulting in the final Spanish version of the instrument, named the Shortened Örebro Musculoskeletal Screening Questionnaire—Spanish Short Form (ÖMSQ-12S), which is presented in Table 2.

### 2.3. Procedure

The study was conducted in accordance with ethical principles for research involving human participants and was approved by the Ethics Committee of Universidad Peruana Unión. Confidentiality, data privacy, and voluntary participation were guaranteed. Before completing the questionnaire, all participants received information about the study objectives and provided informed consent. Data collection was carried out through two modalities: online administration via Google Forms and in-person administration in selected regions. In both cases, response anonymity and the possibility of withdrawing from the study at any time were ensured. Prior to data collection, nursing department heads from health care institutions located in different regions of Peru, including the Coast, Highlands, and Jungle, were contacted. In total, nurses from 14 health care institutions participated. Participants were identified in coordination with health professionals and institutional representatives, prioritizing nurses with musculoskeletal symptoms or complaints related to work activity. This strategy made it possible to evaluate the psychometric behavior of the instrument in a population with direct experience of the phenomenon being assessed. However, no longitudinal outcomes or external indicators such as absenteeism, medical leave, return to work, objective functional disability, or clinical persistence of pain were collected; therefore, the present study does not allow estimation of the instrument’s predictive validity.

### 2.4. Data Analysis

A descriptive analysis of the ÖMSQ-12S items was first performed, considering the mean, standard deviation, skewness, kurtosis, and corrected item-total correlation. Skewness (g_1_) and kurtosis (g_2_) values were considered acceptable when they fell within the ±1.5 range, as an orienting criterion for evaluating the univariate distribution of the items [44]. Likewise, the preliminary quality of the items was examined using the corrected item-total correlation, considering that values less than or equal to 0.20 could indicate a limited contribution of the item to the overall instrument score. Before the factor analyses, items 8, 11, and 12 were recoded using the formula 10 − x, following the instrument’s scoring instructions, so that higher scores represented greater musculoskeletal impairment or risk.

Subsequently, a confirmatory factor analysis (CFA) was performed to evaluate the internal structure of the ÖMSQ-12S. Because most items are answered on numerical scales from 0 to 10, they were treated as approximately continuous variables. The models were estimated using robust maximum likelihood (MLR), a method suitable for correcting possible deviations from multivariate normality and obtaining robust standard errors and fit indices. This decision was supported by the fact that items with multiple response categories can reasonably be analyzed as approximately continuous, especially when they have five or more categories [45,46].

The first model evaluated corresponded to the original three-dimensional structure proposed for the ÖMSQ-12S. However, because modification of factor models and item exclusion in validation studies should not be based solely on statistical criteria, the evaluation of alternative models simultaneously considered overall fit, the magnitude and direction of factor loadings, the conceptual coherence of the items, and the biopsychosocial nature of the instrument. This approach is consistent with recommendations for evaluating measurement properties in health questionnaires, in which interpretability, content validity, and conceptual coherence should be assessed together with psychometric indicators [47].

Accordingly, given the insufficient fit of the original model and the presence of factor loadings in opposite directions within the first factor, an alternative model was estimated in which the functionality/satisfaction items were differentiated from the fear-avoidance items. This decision was theoretically justified because physical functionality and fear-avoidance beliefs are related but conceptually distinguishable components within the biopsychosocial approach to musculoskeletal pain. Likewise, item 1 was evaluated with caution because of its ordinal temporal nature, as it measures the onset or duration of the current pain/problem rather than an intensity of psychological, functional, or symptomatic impairment comparable with most of the items. Therefore, a refined 11-item model was estimated, excluding item 1 from the main factor structure while recognizing its usefulness as complementary descriptive clinical information.

Model fit was evaluated using the robust chi-square statistic (χ^2^), the Comparative Fit Index (CFI), the Tucker–Lewis Index (TLI), the Root Mean Square Error of Approximation (RMSEA) with its 90% confidence interval, the Standardized Root Mean Square Residual (SRMR), and the Akaike Information Criterion (AIC) and Bayesian Information Criterion (BIC). Adequate fit was considered to be indicated by CFI and TLI values equal to or greater than 0.90, preferably close to or greater than 0.95; RMSEA values equal to or less than 0.08, ideally close to 0.06; and SRMR values equal to or less than 0.08 [48,49,50]. Standardized factor loadings (λ) were interpreted as adequate when greater than 0.50 and strong when equal to or greater than 0.70 [49,51].

Convergent validity was evaluated using average variance extracted (AVE), with values equal to or greater than 0.50 considered acceptable, indicating that the construct explains at least 50% of the variance of its indicators [52]. Discriminant validity was examined using the Fornell–Larcker criterion by comparing the AVE of each factor with the squared interfactor correlations (ϕ^2^). Evidence of discriminant validity was considered present when the AVE of each factor was greater than the corresponding ϕ^2^ values.

Internal reliability was evaluated using Cronbach’s alpha coefficient and composite reliability (CR), estimated from the standardized factor loadings. Values equal to or greater than 0.70 were considered acceptable [51,53,54]. All statistical analyses were performed using RStudio (Posit Software, PBC, Boston, MA, USA), an integrated development environment for R software version 4.4.2 (R Foundation for Statistical Computing, Vienna, Austria). Confirmatory factor analyses were estimated using the lavaan package [55]. Statistical significance was set at *p* < 0.05.

## 3. Results

### 3.1. Descriptive Item Statistics

Table 3 presents the descriptive statistics for the ÖMSQ-12S items. Mean scores ranged from 2.62 to 6.24. The item with the highest mean score was item 12, referring to the management of daily routines and social activities after scale reversal (M = 6.24; SD = 3.02), followed by item 11, related to walking for one hour or participating in light recreational activities (M = 6.19; SD = 3.21). The item with the lowest mean score was item 9, referring to the belief that physical activity worsens musculoskeletal pain or problems (M = 2.62; SD = 2.57). Skewness values ranged from −0.41 to 0.78, whereas kurtosis values ranged from −1.72 to 0.37. Overall, these results suggest the absence of extreme deviations from the univariate distribution. However, item 1, referring to the onset or duration of the current pain/problem, showed a kurtosis value of −1.72, indicating a more dispersed distribution of responses. Corrected item-total correlations ranged from 0.18 to 0.75. Item 1 showed the lowest correlation (r = 0.18), anticipating its weaker performance in the confirmatory factor analysis. Reverse-coded items were verified before estimating the factor models.

### 3.2. Confirmatory Factor Analysis

The original three-dimensional model of the ÖMSQ-12S was initially evaluated. This model included three factors: physical activity, fear-avoidance, and satisfaction; psychological aspects; and characteristics of the musculoskeletal problem. Model 1 showed inadequate fit: χ^2^(51) = 276.301, *p* < 0.001, CFI = 0.713, TLI = 0.628, RMSEA = 0.161, 90% CI [0.144, 0.179], SRMR = 0.190, AIC = 8944.680, and BIC = 9066.976. These values indicated that the original three-factor structure did not adequately reproduce the observed relationships among the items. Inspection of the factor loadings showed that the physical activity, fear-avoidance, and satisfaction factor grouped items with divergent empirical behavior. Items 11, 12, and 8, related to functionality and satisfaction, showed positive loadings; however, items 9 and 10, referring to fear-avoidance, showed negative and very low loadings within the same factor (λ = −0.143 and λ = −0.020, respectively). This pattern suggested that the problem did not necessarily lie in the irrelevance of items 9 and 10, but rather in their placement within an overly broad dimension that combined functionality, satisfaction, and fear-avoidance. Likewise, item 1, referring to the onset/duration of the musculoskeletal problem, showed a low loading on the characteristics of the musculoskeletal problem factor (λ = 0.247), indicating a limited contribution as a latent indicator.

Based on these results, Model 2a, an 11-item, four-factor model, was evaluated. In this model, item 1 was excluded, and the original physical activity, fear avoidance, and satisfaction factor was separated into two conceptually distinct dimensions: functioning/satisfaction and fear avoidance. This model was retained as an intermediate step to examine the effect of two psychometric and conceptual decisions: removing item 1 from the main factorial structure due to its poor performance as a latent indicator, and differentiating the fear-avoidance items from the functioning/satisfaction items. Model 2a improved the fit compared with the original model; however, it still showed an elevated RMSEA and a TLI below the recommended criterion: χ^2^(38) = 98.612, *p* < 0.001, CFI = 0.917, TLI = 0.880, RMSEA = 0.097, 90% CI [0.075, 0.119], SRMR = 0.050, AIC = 7829.173, and BIC = 7951.469. Therefore, this model was interpreted as a transitional solution, but not as the most appropriate final structure.

Subsequently, Model 2b was evaluated, retaining the 11-item, four-factor structure while allowing a residual correlation between items 5 and 6. This modification was considered conceptually justifiable because both items assess recent emotional states within the same temporal reference period: tension/anxiety and depression/low mood during the past 2–3 days. Model 2b showed the best overall fit: χ^2^(37) = 58.190, *p* = 0.015, CFI = 0.971, TLI = 0.957, RMSEA = 0.058, 90% CI [0.028, 0.084], SRMR = 0.039, AIC = 7786.552, and BIC = 7911.984. In addition, the robust fit indices for the final model were also adequate: robust CFI = 0.980, robust TLI = 0.970, and robust RMSEA = 0.058. Nevertheless, the residual correlation between items 5 and 6 should be interpreted as a localized adjustment associated with the semantic and temporal proximity of both items, rather than as sufficient evidence that the multidimensional structure has been fully established. Consequently, although Model 2b was retained because of its better fit and conceptual interpretability, its results should be evaluated together with subsequent evidence of convergent and discriminant validity (Table 4).

### 3.3. Factor Loadings of the Original and Refined Models

Table 5 presents the standardized factor loadings of the evaluated models. In Model 1, the loadings were adequate for items 11, 12, and 8 within the first factor, corresponding to physical activity, fear-avoidance, and satisfaction; however, items 9 and 10 showed negative, low-magnitude loadings. This result indicates that the fear-avoidance items did not behave as indicators of the same factor as the functionality/satisfaction items. In the second factor, corresponding to psychological aspects, the loadings were adequate and ranged from 0.639 to 0.822. In the third factor, corresponding to characteristics of the musculoskeletal problem, items 3 and 4 showed high loadings (0.893 and 0.904), whereas item 1 showed a low loading (0.247).

Model 2b included a residual correlation between items 5 and 6. This correlation was statistically significant and moderate in magnitude (r = 0.508, *p* < 0.001), indicating that the items referring to tension/anxiety and depression/low mood share additional specific variance that is not fully explained by the psychological aspects factor. The inclusion of this residual correlation was conceptually defensible because of the semantic and temporal proximity between the two items, as both assess emotional states during the past 2–3 days. However, this modification should be interpreted with caution as a localized model adjustment rather than as a definitive solution to the observed structural limitations. Although no modification indices greater than 10 were observed after including this residual correlation, the interpretation of Model 2b should take into account the limited discriminant validity among some latent dimensions.

### 3.4. Internal Reliability, Convergent Validity, and Discriminant Validity

Table 6 presents the reliability, convergent validity, and discriminant validity indices of the evaluated models. In Model 1, the first factor, corresponding to physical activity, fear-avoidance, and satisfaction, showed low internal reliability (α = 0.593; CR = 0.586) and low convergent validity (AVE = 0.365), confirming that the original dimension combining physical activity, fear-avoidance, and satisfaction did not function adequately as a homogeneous factor. The second factor, corresponding to psychological aspects, showed adequate reliability and convergent validity values (α = 0.828; CR = 0.818; AVE = 0.530). The third factor, corresponding to characteristics of the musculoskeletal problem, showed acceptable composite reliability (CR = 0.759) and adequate AVE (AVE = 0.559), although its alpha was low (α = 0.586), probably influenced by the poor performance of item 1.

In Model 2b, internal reliability was adequate across all factors: functionality/satisfaction (α = 0.801; CR = 0.814), fear-avoidance (α = 0.773; CR = 0.779), psychological aspects (α = 0.828; CR = 0.791), and characteristics of the musculoskeletal problem (α = 0.893; CR = 0.895). These results support the internal consistency of the refined solution.

The convergent validity of Model 2b was adequate for functionality/satisfaction (AVE = 0.603), fear-avoidance (AVE = 0.639), and characteristics of the musculoskeletal problem (AVE = 0.810). The psychological aspects factor showed an AVE slightly below the recommended cutoff of 0.50 (AVE = 0.491), although its composite reliability was adequate (CR = 0.791), suggesting acceptable but not fully optimal convergent evidence for this dimension.

Regarding discriminant validity, Model 2b showed partially satisfactory results. The functionality/satisfaction factor was adequately differentiated from the other factors because the ϕ^2^ values were low relative to fear-avoidance (ϕ^2^ = 0.008), psychological aspects (ϕ^2^ = 0.036), and characteristics of the musculoskeletal problem (ϕ^2^ = 0.029). However, discriminant validity was limited between fear-avoidance and psychological aspects (ϕ^2^ = 0.610), fear-avoidance and characteristics of the musculoskeletal problem (ϕ^2^ = 0.666), and especially between psychological aspects and characteristics of the musculoskeletal problem (ϕ^2^ = 0.960). This latter result substantially exceeded the AVE of both factors, indicating a high degree of empirical overlap between psychological distress and the perception of the musculoskeletal problem in the evaluated sample.

The interfactor correlations of the final model were low between functionality/satisfaction and fear-avoidance (ϕ = −0.087), functionality/satisfaction and psychological aspects (ϕ = −0.190), and functionality/satisfaction and characteristics of the musculoskeletal problem (ϕ = −0.171). In contrast, correlations were high between fear-avoidance and psychological aspects (ϕ = 0.781), fear-avoidance and characteristics of the musculoskeletal problem (ϕ = 0.816), and psychological aspects and characteristics of the musculoskeletal problem (ϕ = 0.980).

### 3.5. Explained Item Variance

Table 7 presents the explained variance of the items in Model 1 and Model 2b. In Model 1, items 9 and 10 showed very low R^2^ values (0.020 and 0.000, respectively), confirming that they were not adequately explained by the original physical activity, fear-avoidance, and satisfaction factor. Likewise, item 1 showed a low R^2^ value (0.061), reinforcing its limited contribution as a factorial indicator of the characteristics of the musculoskeletal problem.

In Model 2b, R^2^ values were adequate for most items. In the functionality/satisfaction factor, values ranged from 0.294 to 0.772; in fear-avoidance, from 0.584 to 0.694; in psychological aspects, from 0.332 to 0.692; and in characteristics of the musculoskeletal problem, from 0.790 to 0.830. Item 8 showed the lowest explained variance within the final model (R^2^ = 0.294), whereas items 3 and 4 showed high values (R^2^ = 0.830 and R^2^ = 0.790, respectively).

### 3.6. Residual Correlation Between Items 5 and 6

Model 2b included a residual correlation between items 5 and 6. This correlation was statistically significant and of moderate magnitude (r = 0.508, *p* < 0.001). This result indicates that the items referring to tension/anxiety and depression/low mood share additional specific variance not fully explained by the psychological aspects factor. The inclusion of this residual correlation was conceptually justifiable because of the semantic and temporal proximity between the two items, as both assess emotional states during the past 2–3 days. In addition, after including this residual correlation, no modification indices greater than 10 were observed in the final model, supporting the stability of the refined solution.

## 4. Discussion

Work-related musculoskeletal disorders constitute a relevant problem among nursing staff due to constant exposure to physical demands, sustained postures, patient mobilization, work overload, and psychosocial factors associated with the health care environment. In this context, having brief, culturally adapted, and psychometrically sound instruments is essential to characterize musculoskeletal risk and guide preventive actions in occupational populations. Therefore, this study aimed to translate, culturally adapt, and examine the structural validity and internal reliability of the abbreviated version of the Örebro Musculoskeletal Screening Questionnaire in Peruvian nurses with musculoskeletal symptoms.

The main finding was that the original three-dimensional structure of the ÖMSQ-12S did not show adequate fit in the evaluated sample. The original model, composed of the following factors—physical activity, fear-avoidance, and satisfaction; psychological aspects; and characteristics of the musculoskeletal problem—showed insufficient fit indices. This result indicates that the initially proposed factor organization did not adequately reproduce the relationships among the items in symptomatic Peruvian nurses. Although the abbreviated version of the ÖMSQ was developed to reduce response burden and facilitate the assessment of injured workers, the results of the present study show that its internal structure may require adjustments when adapted to specific cultural and occupational contexts [40]. One of the main sources of misfit was the first factor of the original model, which grouped functionality/satisfaction items together with fear-avoidance items. In the initial analysis, items 9 and 10, referring to the belief that physical activity worsens pain and the idea of not performing daily routines or work while experiencing pain, showed negative and very low loadings within the original factor. However, when these items were reorganized into an independent fear-avoidance dimension, they showed adequate factor loadings. This finding suggests that items 9 and 10 are not psychometrically irrelevant; rather, they represent a distinct component of the musculoskeletal experience. According to the fear-avoidance model, beliefs about harm, threat, or worsening associated with movement may promote behavioral avoidance, functional restriction, and pain persistence [56,57]. Therefore, separating the fear-avoidance dimension from functionality/satisfaction is theoretically consistent with the biopsychosocial approach to musculoskeletal pain.

The refined 11-item, four-factor solution showed substantially better overall fit than the original model. This model organized the instrument into four dimensions: functioning/satisfaction, fear avoidance, psychological aspects, and characteristics of the musculoskeletal problem. Nevertheless, this structure should be interpreted with caution, because evidence of discriminant validity was limited among some dimensions, particularly between psychological aspects and characteristics of the musculoskeletal problem. Thus, although the four-factor solution offers a theoretically more interpretable organization than the original three-dimensional model, it should not yet be considered a fully established multidimensional structure. Rather, the findings support a preliminary and provisional solution that requires confirmation in subsequent studies with larger and more heterogeneous samples and with external clinical or occupational criteria. This cautious interpretation is consistent with the biopsychosocial approach to musculoskeletal pain, in which somatic, emotional, cognitive, behavioral, and work-related components may substantially overlap in symptomatic populations [58,59].

Another important finding was the poor performance of item 1, referring to the onset or duration of the current pain/problem. This item showed a low factor loading and low explained variance in the original model and was therefore excluded from the refined factor structure. However, its exclusion should not be interpreted as a lack of clinical relevance. Unlike the other items, which assess intensity, functional impairment, emotional distress, or pain-related beliefs, item 1 measures a temporal characteristic using an ordinal scale. Therefore, its psychometric behavior may differ from that of the other items. Clinically, pain duration remains relevant information for contextualizing the course of the musculoskeletal problem; however, in this sample, it functioned better as a complementary descriptive variable than as a latent indicator of a psychometric factor. This interpretation is consistent with the need not to base item removal or retention solely on statistical criteria, but also on conceptual and clinical relevance [47].

The internal reliability of the refined solution was adequate. The functionality/satisfaction, fear-avoidance, psychological aspects, and characteristics of the musculoskeletal problem factors showed Cronbach’s alpha and composite reliability values above 0.70. This indicates acceptable to good internal consistency across all dimensions of the ÖMSQ-11S. In contrast, the original model showed weaknesses in the physical activity, fear-avoidance, and satisfaction factor, as well as in the characteristics of the musculoskeletal problem factor. This improvement supports the reorganization of the items, especially because the original first factor combined heterogeneous content. From a psychometric perspective, a dimension composed of conceptually less homogeneous items tends to show lower reliability and weaker convergent validity, even when some of its items have individual clinical relevance [49,51].

Regarding convergent validity, the functionality/satisfaction, fear-avoidance, and characteristics of the musculoskeletal problem factors exceeded the recommended average variance extracted criterion. This indicates that the retained items explain a sufficient proportion of the variance in their respective constructs. The psychological aspects factor showed an AVE slightly below the 0.50 cutoff, although its composite reliability was adequate. This result suggests that the psychological dimension presents acceptable, though not fully optimal, convergent evidence. One possible explanation is that this factor groups items that, while related, reflect distinct nuances of the psychological and cognitive distress associated with pain, such as anxiety, depressed mood, recovery expectations, and perceived burden in performing daily activities. In symptomatic populations, these manifestations may coexist but not necessarily be expressed with the same intensity across all participants.

Discriminant validity was partially supported. The functionality/satisfaction factor showed adequate differentiation from the other factors; however, high correlations were observed among fear-avoidance, psychological aspects, and characteristics of the musculoskeletal problem. The highest correlation was observed between psychological aspects and characteristics of the musculoskeletal problem, indicating substantial empirical overlap between the two dimensions. This result does not necessarily invalidate the model, but it does require cautious interpretation. In nurses with musculoskeletal symptoms, the intensity or bothersomeness of the physical problem may be closely linked to anxiety, low mood, concern about recovery, and perceived daily burden. From a biopsychosocial perspective, this overlap is clinically plausible, as persistent pain often involves a close interaction between bodily experience, cognitive appraisal, and emotional response [56,59].

### 4.1. Implications

These findings have important implications for the use of the instrument. First, the ÖMSQ-11S should not be interpreted as a definitive version or as a test with confirmed predictive validity. The present study provides evidence of structural validity, partial convergent validity, internal reliability, and an initial assessment of discriminant validity in a symptomatic occupational sample. However, external outcomes such as work absenteeism, medical leave, objective functional disability, pain persistence, or return to work were not evaluated. Therefore, claims about its usefulness for predicting chronicity, disability, or occupational risk should be made with caution. In its current state, the instrument may be considered a structurally refined version for characterizing biopsychosocial dimensions of musculoskeletal problems in symptomatic nurses, but it requires further validation before being recommended as a predictive tool in broad occupational screening programs.

Second, the results suggest that item 1 could be retained as complementary descriptive clinical information, even though it is not part of the ÖMSQ-11S factor score. This decision makes it possible to preserve its clinical utility without compromising the psychometric structure of the refined model. In practical terms, pain duration may remain relevant for classifying the time course of the problem, describing the sample, or exploring associations with clinical variables in future studies. However, its inclusion in the factor score should be reevaluated in future research with larger samples and longitudinal designs.

### 4.2. Limitations

This study has several limitations. First, the sample consisted of nurses with musculoskeletal symptoms, which limits the generalizability of the results to asymptomatic nurses or universal preventive screening programs. This characteristic may have increased the clinical homogeneity of the sample and, potentially, the associations among some dimensions of the instrument. Second, the cross-sectional design prevents assessment of the instrument’s temporal stability; therefore, no evidence of test–retest reliability is available. Third, neither predictive validity nor criterion validity against external clinical or occupational outcomes was evaluated. Fourth, although the refined model showed good fit and adequate reliability, the high correlation between psychological aspects and characteristics of the musculoskeletal problem indicates that the discrimination between these dimensions requires further evaluation.

Future research should examine the performance of the ÖMSQ-11S in larger, more heterogeneous, and representative samples of nurses from different services, regions, and employment conditions. It would also be advisable to include participants with and without musculoskeletal symptoms in order to evaluate its usefulness in preventive occupational screening contexts. Likewise, longitudinal studies are needed to analyze the temporal stability of the instrument and its ability to predict relevant outcomes, such as work absenteeism, functional disability, pain persistence, use of health services, need for physical therapy, or return to work. Finally, additional analyses of factorial invariance by sex, age, region, contract type, or work area would be relevant, as would qualitative studies or cognitive interviews to explore how nurses interpret the fear-avoidance, psychological aspects, and musculoskeletal problem characteristic items.

### 4.3. Conclusions

In conclusion, the present study provides preliminary evidence of structural validity and internal reliability for a refined 11-item version of the ÖMSQ in Peruvian nurses with musculoskeletal symptoms. The original three-factor structure was not supported in the evaluated sample; instead, the four-factor solution showed better overall fit than the original model by distinguishing functioning/satisfaction, fear avoidance, psychological aspects, and characteristics of the musculoskeletal problem. However, this structure should not yet be interpreted as a fully established multidimensional solution, given the limited discriminant validity observed among some dimensions, particularly between psychological aspects and characteristics of the musculoskeletal problem. Therefore, the ÖMSQ-11S should be considered a refined and psychometrically promising, yet still provisional, version. Its use in clinical or occupational settings should be approached with caution, as a complementary descriptive tool rather than as a predictive instrument or universal screening tool, until future longitudinal studies examine its temporal stability, criterion validity, and predictive capacity against external clinical and work-related indicators.

## Figures and Tables

**Table 1:** Descriptive Statistics.

Characteristics		*n*	%
Sex	Female	145	85.3
	Male	25	14.7
Marital status	Married	33	19.4
	Cohabiting	19	11.2
	Divorced	3	1.8
	Single	115	67.6
Educational level	Specialization	39	22.9
	Professional nursing degree	95	55.9
	Postgraduate studies	36	21.2
Employment status	CAS contract	40	23.5
	Contract 728 (indefinite-term)	34	20
	Permanent appointment	36	21.2
	Temporary replacement	15	8.8
	Outsourced employment	45	26.5
Region of origin	Coast	63	37.1
	Jungle	35	20.6
	Highlands	72	42.4

**Table 2:** English and Spanish Versions.

Original Dimension	Item	English Version	Spanish Version
Physical activity, fear-avoidance, and satisfaction/Actividad física, evitación por miedo y satisfacción	11	I can walk for an hour or participate in my normal light recreational or sporting activities	Puedo caminar durante una hora o participar en mis actividades recreativas o deportivas ligeras normales
	12	I manage my daily routine and social activities (e.g., shopping or transport or see friends)	Gestiono mi rutina diaria y actividades sociales (ej. compras, transporte, ver amigos)
	9	Physical activity makes my pain or problem worse	La actividad física empeora mi dolor o problema
	10	I should not do my normal daily routine or work with my present pain or problem	No debería realizar mi rutina diaria normal ni trabajar con mi dolor o problema actual
	8	Think of your life; rate how satisfied you are with your current situation	Piense en su vida; califique cuán satisfecho(a) está con su situación actual
Psychological aspects and others/Aspectos psicológicos y otros	5	During the past 2–3 days, rate how tense or anxious you have felt	Durante los últimos 2–3 días, califique cuán tenso(a) o ansioso(a) se ha sentido
	6	During the past 2–3 days, rate how depressed or down you have felt	Durante los últimos 2–3 días, califique cuán deprimido(a) o desanimado(a) se ha sentido
	7	What do you think is the risk that your current pain or problem will not improve	¿Cuál cree que es el riesgo de que su dolor o problema actual no mejore?
	2	Rate how much of a burden it is to perform all the things you need to do in a normal day	Califique qué tan pesado le resulta realizar todas las actividades que necesita hacer en un día normal
Characteristics of the musculoskeletal problem/Características del problema musculoesquelético	1	When did your current pain or problem start	¿Cuándo comenzó su dolor o problema actual?
	3	For the last 2–3 days, rate on average how bothersome your pain or problem is	En los últimos 2–3 días, califique en promedio qué tan molesto ha sido su dolor o problema
	4	For the last 2–3 days, what percentage of the day do you notice your pain or problem	En los últimos 2–3 días, ¿qué porcentaje del día nota su dolor o problema

**Table 3:** Descriptive Statistics of the ÖMSQ-12S Items.

Original Dimension	Item	M	SD	g_1_	g_2_	r(i-tc)	α
Physical activity, fear-avoidance, and satisfaction	11	6.19	3.21	−0.40	−1.10	0.27	0.82
	12	6.24	3.02	−0.37	−0.94	0.34	0.82
	9	2.62	2.57	0.76	−0.25	0.56	0.82
	10	2.65	2.22	0.66	−0.16	0.46	0.82
	8	5.75	2.66	−0.41	−0.72	0.26	0.82
Psychological aspects and others	5	3.94	2.30	0.49	−0.11	0.58	0.82
	6	3.19	2.80	0.77	−0.37	0.53	0.82
	7	3.14	2.31	0.65	0.36	0.68	0.82
	2	3.00	2.07	0.45	−0.16	0.70	0.82
Characteristics of the musculoskeletal problem	1	5.25	3.76	0.20	−1.72	0.18	0.82
	3	3.18	2.47	0.68	−0.21	0.75	0.82
	4	3.02	2.29	0.78	0.37	0.70	0.82

Note. M = mean; SD = standard deviation; g_1_ = skewness; g_2_ = kurtosis; r(i-tc) = corrected item-total correlation; α = Cronbach’s alpha if the item is deleted. Items 8, 11, and 12 were subsequently recoded using the formula 10 − x.

**Table 4:** Fit Indices of the Evaluated Factor Models.

Model	Structure	χ^2^	df	*p*	CFI	TLI	RMSEA	90% CI RMSEA	SRMR	AIC	BIC
1	ÖMSQ-12S, original three factors	276.30	51	<0.001	0.71	0.63	0.16	[0.144, 0.179]	0.19	8944.68	9066.98
2a	ÖMSQ-11S, four factors, without item 1	98.61	38	<0.001	0.92	0.88	0.10	[0.075, 0.119]	0.05	7829.17	7951.47
2b	ÖMSQ-11S, four factors, without item 1 + oe5~oe6	58.19	37	0.015	0.97	0.96	0.06	[0.028, 0.084]	0.04	7786.55	7911.98

Note. CFI = Comparative Fit Index; TLI = Tucker–Lewis Index; RMSEA = Root Mean Square Error of Approximation; SRMR = Standardized Root Mean Square Residual; AIC = Akaike Information Criterion; BIC = Bayesian Information Criterion.

**Table 5:** Standardized Factor Loadings of the Models Evaluated with the MLR Estimator.

Item	M1			M2			
	F1	F2	F3	F1	F2	F3	F4
11	0.858	—	—	0.863	—	—	—
12	0.882	—	—	0.879	—	—	—
9	−0.143	—	—	—	0.833	—	—
10	−0.020	—	—	—	0.764	—	—
8	0.540	—	—	0.542	—	—	—
5	—	0.693	—	—	—	0.642	—
6	—	0.639	—	—	—	0.576	—
7	—	0.747	—	—	—	0.728	—
2	—	0.822	—	—	—	0.832	—
1	—	—	0.247	—	—	—	—
3	—	—	0.893	—	—	—	0.911
4	—	—	0.904	—	—	—	0.889

Note. M1 = Model 1, original three-factor structure of the ÖMSQ-12S; M2 = Model 2b, refined 11-item, four-factor model. In M1, F1 = physical activity, fear-avoidance, and satisfaction; F2 = psychological aspects; F3 = characteristics of the musculoskeletal problem. In M2, F1 = functionality/satisfaction; F2 = fear-avoidance; F3 = psychological aspects; F4 = characteristics of the musculoskeletal problem. Dashes indicate that the item was not estimated as an indicator of the corresponding factor.

**Table 6:** Reliability, Convergent Validity, and Discriminant Validity of the Models Evaluated with the MLR Estimator.

Parameter	M1			M2			
	F1	F2	F3	F1	F2	F3	F4
AVE	0.365	0.530	0.559	0.603	0.639	0.491	0.810
CR	0.586	0.818	0.759	0.814	0.779	0.791	0.895
α	0.593	0.828	0.586	0.801	0.773	0.828	0.893
ϕ & ϕ^2^							
F1	—	0.038	0.030	—	0.008	0.036	0.029
F2	−0.196	—	0.911	−0.087	—	0.610	0.666
F3	−0.174	0.954	—	−0.190	0.781	—	0.960
F4	—	—	—	−0.171	0.816	0.980	—

Note. M1 = Model 1, original three-factor structure of the ÖMSQ-12S; M2 = Model 2b, refined 11-item, four-factor model with a residual correlation between items 5 and 6. In M1, F1 = physical activity, fear-avoidance, and satisfaction; F2 = psychological aspects; F3 = characteristics of the musculoskeletal problem. In M2, F1 = functionality/satisfaction; F2 = fear-avoidance; F3 = psychological aspects; F4 = characteristics of the musculoskeletal problem. AVE = average variance extracted; CR = composite reliability; α = Cronbach’s alpha; ϕ = interfactor correlation; ϕ^2^ = squared interfactor correlation. In the correlation matrix, interfactor correlations are presented below the diagonal, and their squared values are presented above the diagonal. Discriminant validity is considered adequate when the AVE of each factor is greater than the corresponding ϕ^2^ values.

**Table 7:** Explained Variance of the Items in the Evaluated Models.

Item	R^2^ M1	R^2^ M2
11	0.737	0.744
12	0.778	0.772
9	0.020	0.694
10	0.000	0.584
8	0.292	0.294
5	0.480	0.412
6	0.408	0.332
7	0.559	0.529
2	0.675	0.692
1	0.061	—
3	0.797	0.830
4	0.818	0.790

Note. M1 = Model 1, three-factor ÖMSQ-12S; M2 = Model 2b, four-factor ÖMSQ-11S. R^2^ = proportion of item variance explained by its latent factor. Item 1 was excluded from Model 2b.

## Data Availability

The datasets generated and/or analyzed during the current study are not publicly available due to ethical and privacy considerations but are available from the corresponding author upon reasonable request.
